# Bone adaptation in response to treadmill exercise in young and adult mice

**DOI:** 10.1016/j.bonr.2018.01.003

**Published:** 2018-01-12

**Authors:** Joseph D. Gardinier, Niloufar Rostami, Lauren Juliano, Chunbin Zhang

**Affiliations:** a Bone and Joint Center, Henry Ford Hospital, Detroit, MI 48202, USA; b Wayne State School of Medicine, Detroit, MI 48202, USA

**Keywords:** Bone biomechanics, Exercise, Aging, Sclerostin

## Abstract

Exercise is a key determinate of fracture risk and provides a clinical means to promote bone formation. However, the efficacy of exercise to increase bone mass declines with age. The purpose of this study was to identify age-related differences in the anabolic response to exercise at the cellular and tissue level. To this end, young (8-weeks of age) and adult (36-weeks of age) male mice were subjected to a moderate exercise regimen of running on a treadmill. As a result, exercise had a significant effect on PTHrP and SOST gene expression during the first week that was dependent upon age. In particular, young mice displayed an increase in PTHrP expression and decrease in SOST expression, both of which remained unaffected by exercise in the adult mice. After 5-weeks of exercise, a significant decrease in the percentage of osteocytes expressing sclerostin at the protein level was found in young mice, but not adult mice. Mechanical testing of the tibia found exercise to have a significant influence on tissue-level mechanical properties, specifically ultimate-stress and modulus that was dependent on age. Adult mice in particular experienced a significant decrease in modulus despite an increase in cortical area and cortical thickness compared to sedentary controls. Altogether, this study demonstrates a shift in the cellular response to exercise with age, and that gains in bone mass at the adult stage fail to improve bone strength.

## Highlights

• Cortical bone strength and post-yield properties are enhanced by exercise in young mice, but not adult mice.
• Exercise increases the expression of PTHrP, while suppressing sclerostin expression in young mice, but not adult mice.
• Exercise in adult mice increases bone mass, but fails to increase the mechanical properties of bone.

## 1 Introduction

The aging process predisposes individuals to increased fracture risk due to continual bone loss. As a preventative strategy, exercise and physical activity provide a means to increase peak bone mass in children and adolescents (Greene et al., 2005; Kontulainen et al., 2003; Ward et al., 2005), while allowing adults and elderly to maintain bone mass later in life (Bielemann et al., 2013; Forwood and Burr, 1993; Nikander et al., 2010; Nguyen et al., 2000; Marques et al., 2012; Karlsson, 2002). Despite the ability to maintain bone mass, the capacity to recover bone mass or strength through exercise is extremely limited among older adults (Gomez-Cabello et al., 2012). Clinical studies have reported only modest gains in bone mass that often require exercise regimens with high impact loading that become more and more difficult to perform with age (Karlsson, 2002). In addition, the gain in bone strength following exercise is often limited to vertebrate bodies, while long bones present little to no improvements in fracture rates, especially in the lower limb (Nguyen et al., 2000; Marques et al., 2012; Gomez-Cabello et al., 2012). The minimal gains in bone mass that older adults experience through exercise suggest that aging alters the cellular mechanisms needed to facilitate bone adaptation. However, the specific mechanisms that change with age remain unclear. Understanding how the anabolic response to exercise and physical activity change with age plays key role in developing preventative strategies that can compensate for such deficiencies to promote bone formation in an aging population.

At the tissue level, animal studies have demonstrated during the growth and development phase of rodents that exercise has a positive influence on bone architecture and overall strength. In response to weight-bearing exercises, such as jumping or treadmill running, young mice and rats exhibit increased periosteal bone formation and overall mineral density (Wallace et al., 2007; Kodama et al., 2000; Iwamoto et al., 1999; Iwamoto et al., 2004). While the increase in bone formation due to exercise is considered responsible for increasing the structural-level mechanical properties of bone, the coinciding increase in tissue-level mechanical properties and fracture toughness have been attributed to changes in both the mineral and matrix composition (Kohn et al., 2009; Gardinier et al., 2016; Hammond et al., 2016; McNerny et al., 2015; Wallace et al., 2010). Although a few studies have demonstrated similar adaptations in mice that have reached skeletal maturity, (which occurs around 16-weeks of age), the effect that exercise has on tissue adaptation after skeletal maturity is reached has yet to be evaluated (Kohn et al., 2009; Bennell et al., 2002; Gardinier et al., 2015). To simulate dynamic loading during exercise, exogenous loading models have been used to demonstrate that aged mice require larger strains to invoke bone formation that younger mice experience at lower strains (De Souza et al., 2005; Meakin et al., 2014; Brodt and Silva, 2010; Lynch et al., 2011). Based on in-vivo loading studies alongside clinical observations, the cellular mechanisms that regulate the mechanostat of bone appear to shift with age (Turner et al., 1995).

At the cellular level, the anabolic response to exercise is considered a function of different stimuli, most notably the dynamic loading and systemic changes in calcitropic hormones, such as parathyroid hormone (PTH) (Gardinier et al., 2015). Bone remodeling in response to both mechanical loading and PTH is largely facilitated by osteocytes' activation of osteoblasts and osteoclasts through the release of various secondary messengers (Bellido, 2014). In particular, osteocytes release the receptor activator of nuclear factor kappa-B ligand (RANK-L) and its inhibitor osteoprotegerin (OPG) to regulate osteoclast activity. To activate osteoblasts, osteocytes suppress the Wnt inhibitors sclerostin and dickkopf-related protein-1 (DKK1), while releasing various nucleotides, prostaglandins, cytokines, and hormones, such as the PTH-related protein (PTHrP). In young animal models direct loading as well as exercise has been shown to reduce sclerostin expression (Gardinier et al., 2016; Robling et al., 2008; Tu et al., 2012), while clinical findings have demonstrated that physical activity tends to lower sclerostin and increase OPG levels in circulation (Mezil et al., 2015; Ardawi et al., 2012; Zagrodna et al., 2016; Amrein et al., 2012). However, mixed findings have been reported regarding the influence that exercise has sclerostin levels as well as RANK-L and OPG in older populations (Amrein et al., 2012; Bergstrom et al., 2012; Marques et al., 2013; Vincent and Braith, 2002; Gombos et al., 2016). At the same time, animal studies have yet to identify age-related differences in the underlying mechanisms that facilitate bone adaptation in response to exercise.

Altogether, the specific mechanisms that change with age and effectively attenuate the anabolic potential of exercise have yet to be established. We hypothesized that the capacity to suppress sclerostin at the gene and protein level in response to exercise is lost with age, coinciding with failure to enhance bone formation and the mechanical properties of cortical bone. To test this hypothesis, we developed an exercise model that used 8- and 36-week old male mice alongside a moderate exercise regimen of running on a treadmill. Alterations in the anabolic response were then evaluated at the tissue and cellular level.

## 2 Methods

### 2.1 In-vivo protocols

All animal procedures were performed at the Henry Ford Hospital under approval from the Institutional Animal Care Use Committee (IACUC). Male C57Bl/6J mice were purchased from Jackson Laboratories (Bar Harbor, ME), and housed with sufficient water, food, and items for enrichment. At 8-weeks and 36-weeks of age, mice were divided into three weight-matched groups: baseline, sedentary, and exercise. Each exercise group was subjected to running on a treadmill (Columbus Instruments, OH) at a 5° incline and speed of 15 m/min for 30 min, five days per week. After the first week of exercise, a subset of 6 mice from each sedentary and exercise group were euthanized to collect mRNA samples from both tibiae to evaluate gene expression. The remaining mice in each group received 2 fluorochrome injections on day 2 (alizarin complexone, 25 mg/kg) and day 31 (xylenol orange, 90 mg/kg) to quantify the degree of mineralization and apposition rate during the course of the experiment. After 5-weeks of exercise, mice from both sedentary and exercise groups were euthanized. The right and left tibia were collected from 10 mice for mechanical testing, micro-CT analysis, and histomorphometry, while another 5 right and left tibia were collected for immunohistochemistry. Baseline samples were collected in the same manner at 8-weeks and 36-weeks of age. Based on our previous studies, sample size was selected in order to detect a 15% difference in tissue strength with a power of 80% and significance level of 0.05 (Gardinier et al., 2016; Gardinier et al., 2015).

### 2.2 Gene expression

Tibia samples used to evaluate initial changes in gene expression were cleaned of excess soft tissue and both epiphyses before removing the bone marrow through centrifugation to obtain ‘osteocyte-enriched’ samples as described previously (Kelly et al., 2014). Each sample was then immediately frozen in liquid nitrogen and homogenized to extract mRNA with Trizol. The RNeasy® Mini Kit (Qiagen, Hilden, Germany) was used to purify the mRNA prior to generating cDNA with the Taqman cDNA synthesis kit (Applied Biosystems, CA). The qRT-PCR was then carried out using an Applied BioSystems 7500 RealTime PCR machine along with the following Taqman primers: GAPDH (Mm99999915), RANK-L (Mm00441906), OPG (Mm00435454), SOST (Mm00470479), DKK1 (Mm00438422), and PTH-related protein (Mm00436057). All samples were analyzed in triplicates and normalized to their respective expression of GAPDH using the 2^-ΔΔct^ method, and then normalized again to 8-week controls.

### 2.3 Micro-CT analysis

Cortical bone architecture was derived from ex-vivo scans of the entire tibia using a custom-built μCT system previously described (Reimann et al., 1997). Each tibia is scanned with the following settings: 14 μm voxel size, 60 kVp, 0.5 mm aluminum filter, 83 μA, and 720 views over 360°, with each view averaging 4 frames. Images were reconstructed using a greyscale threshold optimized across all the samples. Images of each tibia were oriented to match their position during mechanical testing. The fracture site was defined by measuring where the crack initiated along the medial side under tension relative to the distal tibia- fibula junction. A standard site was also defined midway between the loading points. At both sites, cortical bone thickness, cross-sectional area, endosteal surface, periosteal surface, bone mineral content (BMC), bone mineral density (BMD), distance from the most lateral surface to the neutral axis, and moment of inertia about the anterior–posterior axis (MOI_A/P_) were determined. The BMD was determined based on grey scale values converted into grams K_2_HPO_4_ per cm^3^ using a calibration curve of liquid standards as described in literature (Nazarian et al., 2008).

### 2.4 Mechanical testing

The mechanical properties of the tibia were measured under four-point bending using the EnduraTech ELF 3200 Series (Bose®, MA). The base supports spanned 9 mm, while the loading points spanned 3 mm. Each tibia was loaded until failure at a rate of 0.025 mm/s with the lateral surface position under compression. Load and displacement were recorded during testing, and the location of failure was measured relative to the tibia-fibula junction. Structural-level properties were then quantified based on the force–deflection curve (yield-deformation, yield-load, stiffness, ultimate-load, post-yield displacement, and post-yield work). Tissue-level properties were estimated using the following beam-bending equations to calculate stress and strain:

Stress = σ = Fac/2I_AP_

Strain = ε = 6 cd/a(3L − 4a)where “F” is the recorded force, ‘a’ is the distance between loading supports, ‘c’ is the distance from the medial surface to the neutral axis, I_AP_ is the moment of inertia along the anterior-posterior axis, ‘d’ is the measured displacement, and ‘L’ is the span between the outer supports.

### 2.5 Histomorphometry

Fluorochrome labels marking mineralization were quantified by first embedding each tibia sample in methyl methacrylate (Koldmount Cold Mount kit, Mager Scientific, MI) following a graded ethanol dehydration. Sections were then cut at the mid-diaphysis using a low-speed sectioning saw (South Bay Technology, Model 650, CA) with a diamond wafering blade (Mager Scientific, MI), and then polished on wet silicon carbide abrasive disks to a final thickness of 200 μm. Fluorochrome labels were identified under a confocal fluorescence microscope (FLUOVIEW FV1000, Olympus) to calculate the mineralizing surfaces (MS/BS) as a percentage of the endosteum and periosteum surfaces alongside the mineral apposition rate (MAR) and bone formation rate (BFR) according to standardized histomorphometric analysis using ImageJ software (Dempster et al., 2013). Instances of only single labeling were assigned a MAR value of 0.1 μm/day as described in literature (Qiu et al., 2017).

### 2.6 Immunohistochemistry

To evaluate changes in protein expression, tibia samples were fixed in 4% paraformaldehyde and demineralized in 10% acidic acid before being embedded in paraffin. 5 μm thick cross-sections were cut at the mid-diaphysis of the tibia. Each section was deparaffinized in xylene, rehydrated through graded ethanol, and then washed with 0.3% hydrogen peroxide to remove endogenous peroxidases. Primary antibodies for sclerostin (AF1589, R&D Systems Inc., MN) or PTH-related protein (B6787, LifeSpan BioSciences Inc., WA) were diluted at 1:200 in PBS with 10% donkey serum (Jackson ImmunoResearch Inc., PA) before being applied over-night at 4 °C. The primary antibodies were then detected with biotin-conjugated secondary antibodies and a diaminobenzidine (DAB) substrate using the Vectastain Elite ABC kit (Vector Labs, Inc., CA) according to the manufacturer's instructions. The number of sclerostin positive and total osteocytes were quantified and averaged across 5 samples within each group.

### 2.7 Statistical analysis

A student t-test was used to identify significant differences in cortical bone architecture and mechanical properties of bone between 8-week and 36-week old baseline controls. For all non-baseline groups, statistical differences were identified for each outcome measure using a two-way analysis of variance (ANOVA) with Tukey post-hoc testing to identify interactions between specific groups. Statistical significance under each analysis was defined by a p-value < 0.05. For each measure, the group mean and standard deviations were reported.

## 3 Results

### 3.1 Exercise has largest effect on cortical bone architecture in adult mice

**Fig. 1:**
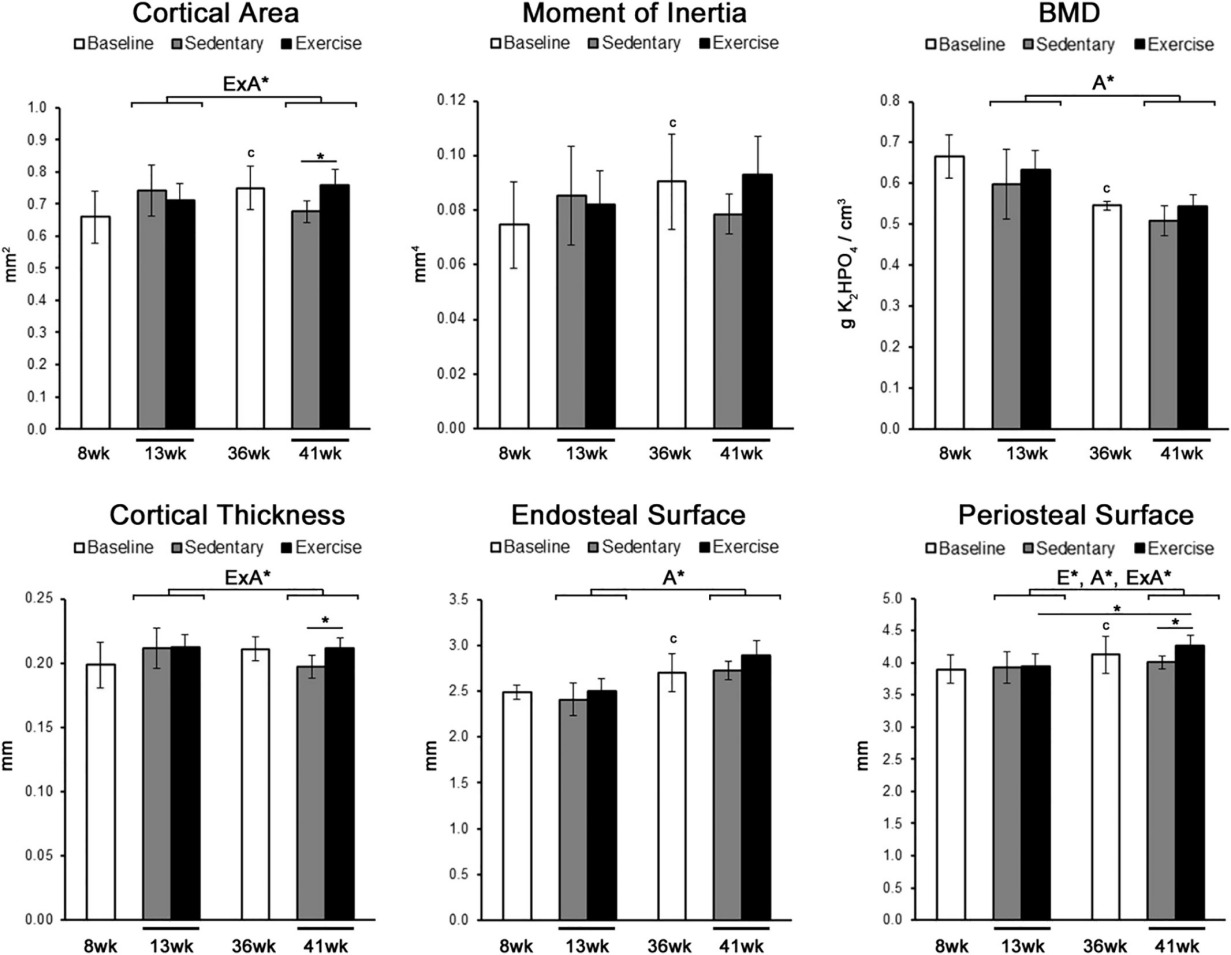
Exercise had the largest effect on cortical bone architecture in adult mice. Cortical bone architecture was measured at the mid-diaphysis of the tibia using micro-CT analysis. The moment of inertia was calculated along the anterior–posterior axis. Significant main factor effects are noted for age (A*), exercise (E*), and their interaction (ExA*). Tukey post hoc analysis between sedentary and exercise with a p-value < 0.05 are noted by ‘*’, while student t-tests between baseline groups with a p-value < 0.05 are noted by ‘c’. Mean ± std. are shown (n = 10).

Based on micro-CT analysis of the tibia, the cortical area and moment of inertia at baseline was significantly larger in 36-week old mice compared to 8-week old mice, while BMD was significantly smaller in 36-week old mice (Fig. 1). The increase in moment of inertia and cortical area with age corresponded with an increase in the endosteal and periosteal perimeters. Results from a two-way ANOVA found exercise had a significant effect on cortical area, cortical thickness, and the periosteal perimeter that were dependent on age. In particular, exercised 41-week old mice had a significantly larger cortical area and cortical thickness compared to 41-week old sedentary controls due to a significant increase in the periosteal perimeter. Such changes in architecture were not found in exercised 13-week old mice.

Histomorphometry analysis demonstrated that age also had a main effect on the MS/BS, causing a significant decrease along both endosteum and periosteal surfaces in 41-week old mice. The interaction between exercise and age had a significant effect on the MAR at the endosteal surface and periosteal surface. In addition, BFR at the periosteal surface also displayed a significant interaction between age and exercise. At the periosteal surface in particular, exercise significantly increased both MAR and BFR in 13-week old mice compared to 13-week old sedentary controls as well as 41-week old exercised mice.

### 3.2 Exercise improves bone strength in young mice, but not adult mice

**Fig. 2:**
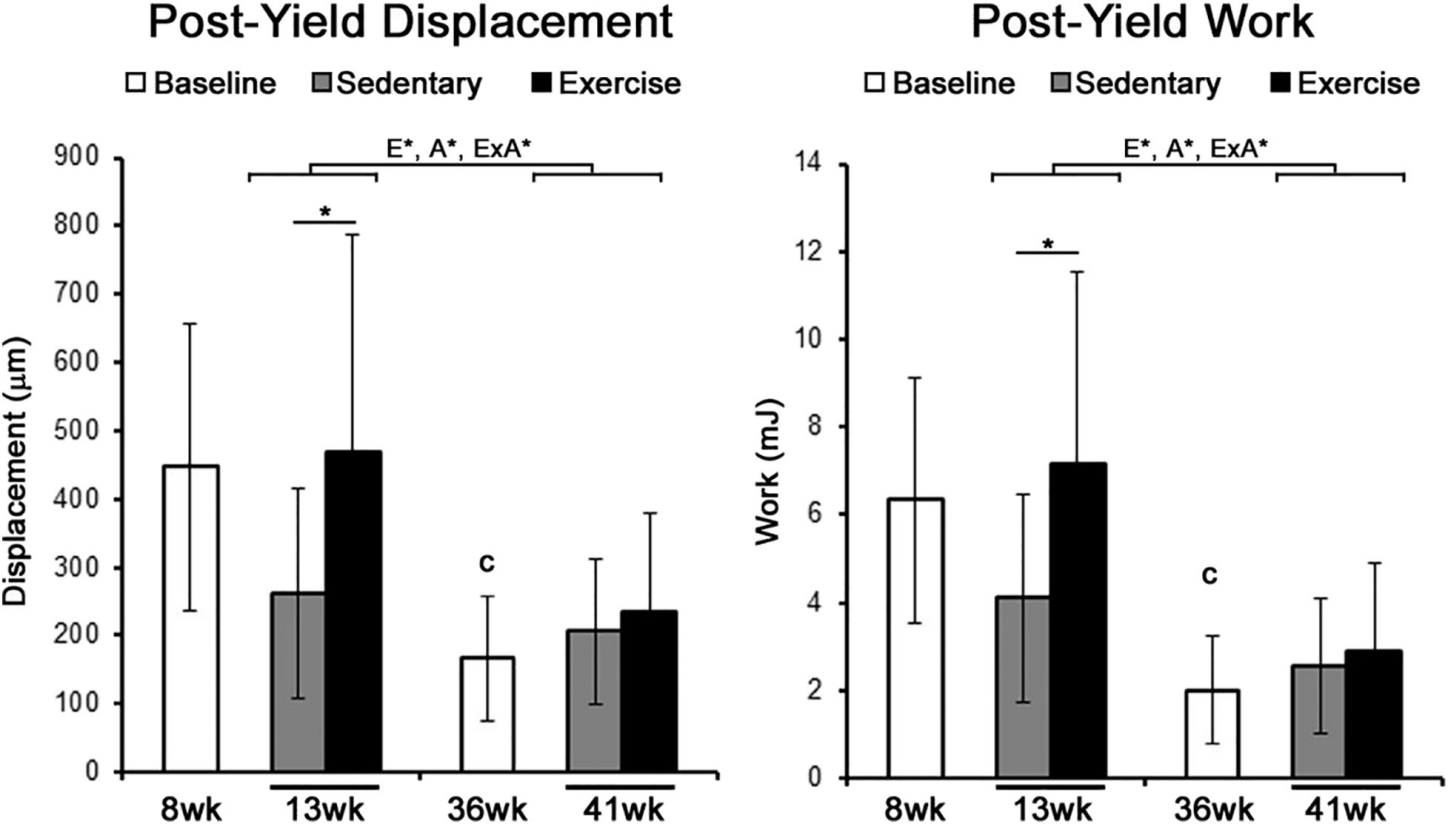
The post-yield behavior of the tibia after 1-week of exercise was significantly increased in younger mice. The post-yield displacement and work of the tibia were measured under four-point bending. Significant main factor effects are noted for age (A*), exercise (E*), and their interaction (ExA*). Tukey post hoc analysis between sedentary and exercise with a p-value < 0.05 are noted by ‘*’, while student t-tests between baseline groups with a p-value < 0.05 are noted by ‘c’. Mean ± std. are shown (n = 10).

At baseline, mechanical properties of the tibia from 36-week old mice exhibited significantly smaller ultimate-load, ultimate-displacement, yield-stress, yield-strain, ultimate-stress, and ultimate-strain (Table 2). The post-yield displacement and post-yield work at baseline were significantly smaller in 36-week old mice compared to 8-week old mice (Fig. 2).

Results from the two-way ANOVA found that the effect of exercise on the modulus, ultimate stress, and both post-yield displacement and post-yield work to be significantly dependent upon age (Table 2 & Fig. 2). Based on the post-hoc analysis, 13-week old mice that were exercised had a significantly greater ultimate stress and post-yield properties compared to 13-week old sedentary controls. Conversely, 41-week old mice that were exercised had a significantly smaller modulus compared to 41-week old sedentary controls. Across both sedentary and exercise groups, post-hoc analysis found age had a significant effect on the yield-load and ultimate-load, while the main effect of age on yield-displacement and stiffness were driven by significant differences across just exercise groups.

### 3.3 Exercise fails to invoke anabolic gene expression in adult mice

**Fig. 3:**
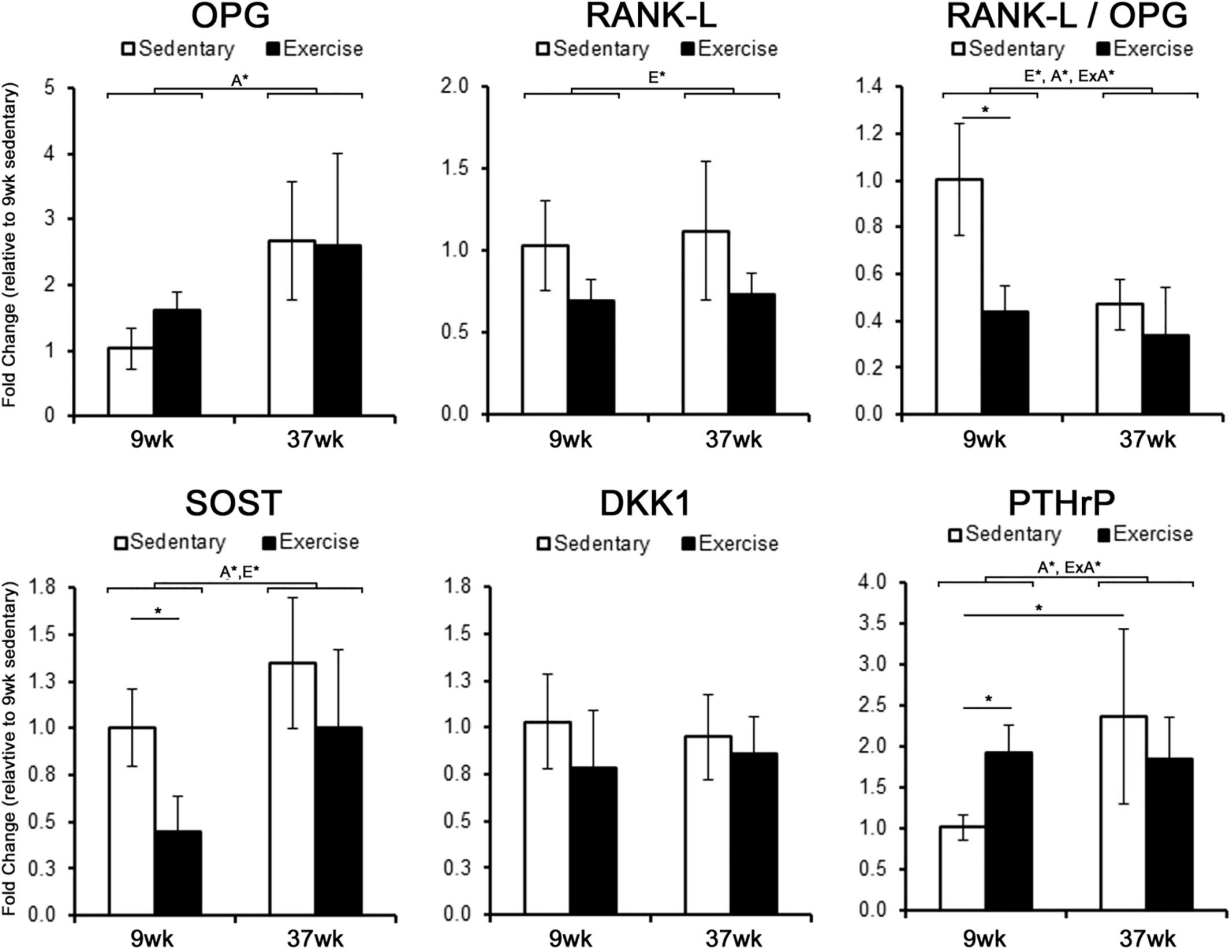
Initial changes in gene expression following 1-week of exercise are attenuated in adult mice. The fold change in mRNA expression within cortical bone was measured after 5-days of exercise and expressed relative to 13-week sedentary controls. Significant main factor effects are noted for age (A*), exercise (E*), and their interaction (ExA*). Tukey post hoc analysis between sedentary and exercise with a p-value < 0.05 are noted by ‘*’. Mean ± std. (n = 6).

After 1-week of exercise, gene expression within cortical bone of the tibia was evaluated to identify changes in cell signaling mechanisms that initiate and promote bone adaptation. Bone resorption in particular is dependent upon osteoclast recruitment through OPG and RANK-L signaling. Based on mRNA levels, age had a significant effect on OPG expression, causing a 2.5 fold increase at 37-weeks of age (Fig. 3), while exercise had a significant effect on RANK-L expression, causing a 30% decrease in expression. A significant interaction between exercise and age was found for the resulting RANK-L/OPG ratio, with 9-week old mice exhibiting a significant decrease in the RANK-L/OPG ratio that was not present in 37-week old mice. Furthermore, we also found age and exercise had a main effect on SOST expression, but not DKK1. Post-hoc analysis found SOST expression significantly smaller in 9-week old mice after exercise compared to sedentary controls. Conversely, 41-week old mice subjected to exercise displayed no significant difference in SOST expression compared to 41-week old sedentary controls. Given that SOST expression during exercise is influenced by activation of the PTH/PTHrP receptor (Gardinier et al., 2016), the expression of the PTHrP in cortical bone was also measured. Age alone had a main effect on PTHrP expression, with a 2.1 fold increase between 9-week and 37-week old sedentary mice being significant. In addition, there was a significant interaction between exercise and age, with exercise causing a significant increase in PTHrP expression in 9-week old mice but not 37-week old mice when compared to their respective sedentary controls.

**Fig. 4:**
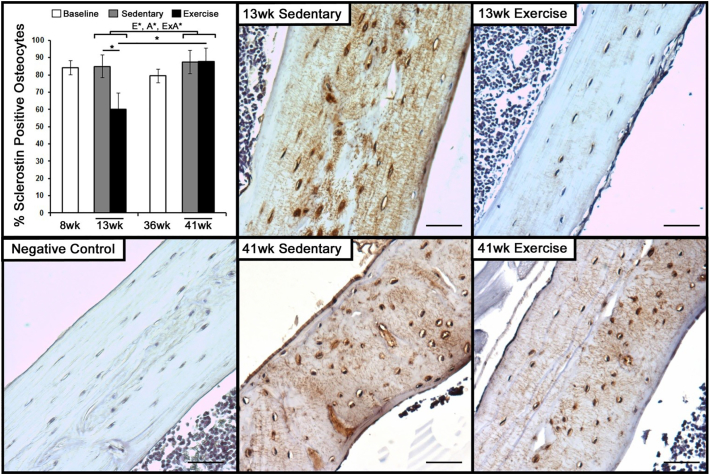
Exercise decreases the expression of Sclerostin among osteocytes in young mice, but not adult mice. The number of osteocytes and sclerostin-positive osteocytes were measured in 8-week and 36-week old baseline controls, along with 13-week and 41-week old mice that were exposed to 5-weeks of exercise or sedentary conditions. Significant main factor effects are noted for age (A*), exercise (E*), and their interaction (ExA*). Tukey post hoc analysis between sedentary and exercise with a p-value < 0.05 are noted by ‘*’. Representative images of immunohistochemistry for sclerostin in cortical bone of the tibia following 5-weeks of sedentary or exercise conditions in 8-week and 36-week old mice. Bar indicates 100 μm.

Given the initial changes in gene expression, subsequent changes in protein expression were then evaluated after 5-weeks of exercise. Immunohistochemistry revealed that the presence of sclerostin is lost throughout cortical bone in 13-week old mice that were exercised, with the number of osteocytes positively stained for sclerostin significantly decreased compared to sedentary controls (Fig. 4). Staining for sclerostin in 13-week old sedentary mice was localized to the lacuna as well as the canaliculi. In contrast, exercise had no effect on the presence of sclerostin throughout cortical bone or number of osteocytes positive for sclerostin in 41-week old mice.

**Fig. 5:**
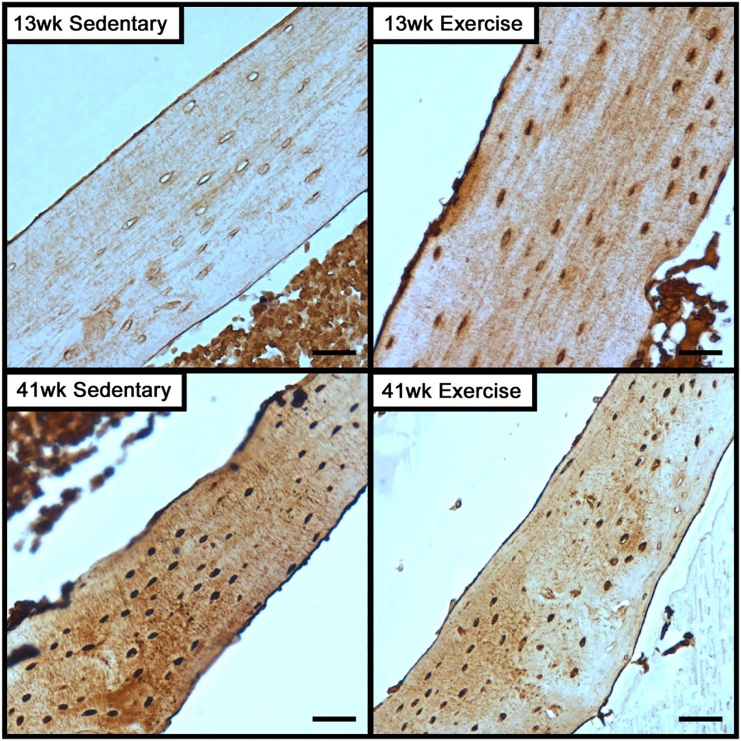
PTHrP expression in cortical bone is high among adult mice, but does not change with exercise as in younger mice. Representative images of immunohistochemistry for PTHrP expression in cortical bone of the tibia following 5-weeks of sedentary or exercise conditions. Bar indicates 100 μm.

Immunohistochemistry also revealed a greater presence of PTHrP in 41-week old sedentary mice compared to 13-week old sedentary mice (Fig. 5). In 13-week old mice that were subjected to exercise, staining of PTHrP was more pronounced throughout cortical bone. Staining of PTHrP was primarily localized to the osteocytes, with relatively lower degree of staining in the canaliculi. In 41-week old mice, exercise appears to have little impact on the presence of PTHrP throughout cortical bone compared to 41-week old sedentary controls.

## 4 Discussion

The present study demonstrates that exercise in adult mice fails to improve the mechanical strength of bone despite relative gains in bone mass. In clinical studies, adults who undertake various forms of exercise, such as running, experience little improvement in bone quality (Forwood and Burr, 1993; Nikander et al., 2010; Gutin and Kasper, 1992; Bailey and McCulloch, 1990). Exercise in general is noted for maintaining bone mass, while providing only modest gains in BMD (Karlsson, 2002). For example, Forwood et al. concluded that at best impact exercise can produce a 1–3% increase in BMD, which is not sufficient to reduce fracture risk (Forwood and Burr, 1993). In agreement, our findings demonstrate that the mechanical properties of bone in adult mice do not improve with exercise, despite increased cortical area and cortical thickness compared to sedentary controls. Conversely, exercise during the growth and development phase of young mice increased bone strength (ultimate-stress) and post-yield behavior (displacement and work), without requiring significant changes in bone mass or architecture. At the cellular level, exercise in adult mice also failed to elicit initial changes in PTHrP and SOST/sclerostin expression that young mice experience. In particular, the inability to suppress sclerostin presents a potential limitation for exercise to promote bone formation and improve bone strength in adults. Altogether, these data indicate that exercise in adult mice fails to improve the mechanical properties of bone, and that this lack of adaptation is related to a loss in key mechanotransduction pathways.

Consistent with previous studies, exercise improved the post-yield properties of bone compared to sedentary mice (McNerny et al., 2015; Wallace et al., 2010; Gardinier et al., 2015; Wallace et al., 2009). In particular, exercise increased the post-yield deformation and post-yield work of bone, indicating a greater capacity to absorb energy under loading. The gain in energy absorption and post-yield behavior through during exercise was also unique to young mice and not adults that have long ceased to actively grow. The capacity for bone to absorb energy under loading is largely defined by the inherent properties of the collagen matrix, such as cross-linking profiles, as well the presence of non-collagenous proteins (Wallace et al., 2010; Burstein et al., 1975; Morgan et al., 2015). Although a direct relationship has not been established, liquid chromatography and vibrational spectroscopy have identified key shifts in the collagen cross-linking profile of growing and adolescent mice following treadmill exercise (Kohn et al., 2009; Hammond et al., 2016; McNerny et al., 2015). For example, McNerny et al. demonstrated that exercise starting at 5-weeks of age shifts the maturation of collagen cross-links toward the pyridinoline pathway and that the accumulation of pyridinolines influenced the mechanical behavior of bone. The extensive turnover and formation of new matrix allows exercise to have a significant impact on collagen maturation during growth and development. However, as mice age and bone turnover subsides, the capacity to then shift collagen maturation through exercise is likely to diminish, limiting the efficacy of exercise to improve the post-yield properties in adult mice.

As expected, younger mice displayed a higher degree of bone formation than adult mice, specifically at the periosteal surface where both MS/BS and MAR significantly decreased with age (Table 1). In response to exercise, younger mice displayed an increase in periosteal MAR and BFR, both of which significantly decreased with age. In a similar manner, exogenous loading models have reported an increase in periosteal formation that declines with age (Brodt and Silva, 2010; Lynch et al., 2011; Srinivasan et al., 2003). However, the increase in bone formation during exercise did not correspond with an increase in periosteal expansion that is typical of exogenous loading. Furthermore, adult mice exhibited an increase in cortical area and periosteal expansion despite a lack of formation. Although the reason for these discrepancies is unclear, it is likely that changes in bone resorption due to exercise would influence the net change in cortical architecture. Un-like the pure anabolic response to exogenous loading, exercise elicits a catabolic response that is transient in nature, with bone resorption markers such as cross-linking propeptide and RANK-L increased within the first hour after exercise before returning to a baseline (Mezil et al., 2015; Guillemant et al., 2004; Scott et al., 2010; Welsh et al., 1997; Langberg et al., 2000). Mezil et al. went on to demonstrate in young adults (23 years of age) that protein levels of RANK-L drop below baseline (Mezil et al., 2015), suggesting a new set point that could be explained by the decrease in RANK-L expression we observed at the gene level (Fig. 3). The catabolic activity that exercise initiates has been attribute to the increase in systemic parathyroid hormone (PTH) (Guillemant et al., 2004; Scott et al., 2010), a condition that exogenous loading models do not mimic. Unfortunately, the protein levels of RANKL and degree of resorption were not monitored during this study, presenting a key limitation in discerning the effects of exercise on bone turnover. Further investigation is warranted to understand how exercise influences catabolic activity at each age group, and its particular interaction with the anabolic response.

**Table 1:** Histomorphometry of the mouse tibia following 5 weeks of exercise.

	13 weeks		41 weeks		Main effects		
	Sedentary avg (std.)	Exercise avg (std.)	Sedentary avg (std.)	Exercise avg (std.)	Age (p)	Ex (p)	Int (p)
Endosteal properties							
MS/BS (μm/μm)	0.39 (0.25)	0.43 (0.23)	0.22 (0.11)	0.32 (0.12)	<0.05		
MAR (μm/day)	1.52 (1.50)	0.80 (0.79)	0.55 (0.50)	1.73 (1.70)			<0.05
BFR (μm/μm/day)	0.61 (0.59)	0.53 (0.52)	0.17 (0.15)	0.63 (0.61)			

Periosteal properties							
MS/BS (μm/μm)	0.33 (0.14)	0.44 (0.15)	0.26 (0.09)	0.25 (0.15)	<0.05		
MAR (μm/day)	0.24 (0.23)	1.85 (1.22)^b^	0.24 (0.22)	0.52 (0.49)^a^	<0.05	<0.001	<0.05
BFR (μm/μm/day)	0.10 (0.11)	1.01 (0.60)^b^	0.06 (0.08)	0.21 (0.20)^a^	<0.05	<0.01	<0.05

n = 10, Ex = Exercise, Int = Interaction between exercise and age, n.s. = not significant.

a p-value < 0.05 compared to 8-week control.

b p-value < 0.05 compared to sedentary control.

**Table 2:** Mechanical Properties of the mouse tibia following 5-weeks of exercise.

	8 weeks	13 weeks		36 weeks	41 weeks		Main effects		
	Baseline avg (std.)	Sedentary avg (std.)	Exercise avg (std.)	Baseline avg (std.)	Sedentary avg (std.)	Exercise avg (std.)	Age (p)	Ex (p)	Int (p)
Structural-level properties									
Yield load (N)	13.5 (3.0)	16.1 (3.3)	18.2 (3.3)	12.4 (3.5)	11.5 (2.7)^a^	12.0 (3.2)^a^	<0.001		
Yield displacement (μm)	240.6 (68)	238.8 (41)	261.0 (74)	214.7 (34)	196.8 (40)	211.9 (72)^a^	<0.01		
Ultimate load (N)	16.6 (3.5)	17.6 (2.9)	19.6 (3.2)	13.1 (3.3)^c^	13.5 (3.2)^a^	13.9 (2.6)^a^	<0.001		
Ultimate displacement (μm)	392.8 (95)	330.9 (125)	335.1 (109)	254.1 (38)^c^	302.0 (66)	285.7 (61)			
Stiffness (N/mm)	70.6 (18)	83.4 (19)	87.1 (22)	69.5 (16)	75.5 (16)	67.8 (19)^a^	<0.05		

Tissue-level properties									
Yield stress (MPa)	164.9 (31)	167.2 (51)	205.2 (49)	110.5 (40)^c^	117.2 (34)^a^	97.8 (40)^a^	<0.001		
Yield strain (με)	23,804 (5208)	22,732 (4007)	25,668 (7091)	20,195 (3581)^c^	18,273 (3363)	21,327 (8007)	<0.01	<0.05	
Ultimate stress (MPa)	203.9 (36)	183.9 (42)	221.1 (50)^b^	117.6 (41)^c^	136.8 (39)^a^	112.6 (36)^a^	<0.001		<0.05
Ultimate strain (με)	39,664 (9827)	31,582 (10,385)	33,361 (12,256)	23,929 (4316)^c^	28,268 (6737)	28,733 (6820)			
Modulus (GPa)	8.65 (2.2)	9.14 (3.1)	9.79 (3.3)	6.62 (2.3)	8.23 (2.3)	5.39 (1.4)^b^	<0.01		<0.05

n = 10, Ex = Exercise, Int = Interaction between exercise and age.

a p-value < 0.05 compared to 13-week old control.

b p-value < 0.05 compared to sedentary control within same age-group;

c p-value < 0.05 of student t-test compared to 8-week baseline group.

Osteocytes' down-regulation of the Wnt inhibitors sclerostin and DKK1 play a key role in facilitating osteoblast activation (Burgers and Williams, 2013). In young mice, exercise had the largest effect on the SOST gene within the first week, while our previous studies have even found SOST expression to decrease within the first two days of exercise (Gardinier et al., 2016). The initial decrease in SOST expression was then followed by a significant decrease in sclerostin expression at the protein level throughout the cortical bone. In contrast, adult mice demonstrated a lack of SOST/scelrostin suppression in response to exercise at the gene and protein level. Even though men and women who are more active tend to display lower circulating levels of sclerostin (Amrein et al., 2012), the efficacy of exercise to suppress sclerostin has varied between studies (Bergstrom et al., 2012; Armamento-Villareal et al., 2012; Hinton et al., 2017; Grasso et al., 2015; Kerschan-Schindl et al., 2015). Similarly to our findings, long-term studies found no significant changes in sclerostin levels after 1 year of intervention (Bergstrom et al., 2012; Armamento-Villareal et al., 2012). The inability to suppress sclerostin during exercise suggests a shift in the osteocytes' functional response to exercise.

Osteocytes' expression of sclerostin is heavily influenced by mechanical loading and activation of the PTH/PTHrP receptor (PPR) (Bellido et al., 2013; Uda et al., 2017). Blocking PPR activation during exercise has been shown to block SOST suppression alongside changes in mechanical behavior (Gardinier et al., 2016). Although PPR activation in response to exercise is often attributed to the release of PTH from the parathyroid glands, the present study demonstrates a potential contribution from PTHrP expression within bone. Based on in-vitro studies, dynamic loading produces a transient release of PTHrP from osteocyte-like cell lines (Maycas et al., 2015). However, loading under our exercise regimen had no effect on PTHrP expression in adult mice, both at the protein and gene level. As a result, the inability to increase PTHrP may explain why SOST expression remained relatively high in response to exercise. Given that PPR activation during exercise contributes to bone adaptation, the autocrine and paracrine function of PTHrP during exercise warrants further investigation regarding its particular regulation of SOST/sclerostin and their influence on bone quality.

Age-related changes in the cellular response to exercise may extend from several factors, such as differences in the applied strain or changes in the cell behavior. In particular, the tissue modulus of bone can change with age, altering the magnitude of strain generated at the surfaces under a given load. For example, 26-week old mice have been shown to experience twice as high strains as those generated in 10-week old mice under a given load (Lynch et al., 2011). In addition, the force required to produce ~2200 μ-strains along the tibia of mice decreases with age (Holguin et al., 2014). Given the differences in size, muscle strength, and ground reaction forces during running, we speculate that 36-week old mice will experience greater tibial strains than 8-week mice. Therefore, the lack of response to exercise is not likely to be a function of less tissue strain. The need for higher strains to elicit an anabolic response in aged mice suggests a fundamental change in cell behavior that warrants further investigation.

Overall, our findings demonstrate using mice that exercise increases the post-yield behavior of cortical bone during growth and development. In adult mice, exercise increases bone mass without improving cortical bone strength, but at the expense of tissue modulus. Furthermore, aging attenuates osteocytes' capacity to suppress sclerostin and increase PTHrP expression in response to exercise, both of which present a limitation in the anabolic potential of exercise among adults.
